# Intact glycoconjugates from *Taenia crassiceps* excreted/secreted products ameliorate chemically induced colitis by modulating inflammation and strengthening adherens junctions

**DOI:** 10.1007/s10787-025-01821-y

**Published:** 2025-06-27

**Authors:** Yadira Ledesma-Soto, Ilse Chávez-Soto, Marissa Calderón-Torres, Andrea Monserrat Rodríguez-Lozoya, Jonadab E. Olguin, Luis B. Hernández-Portilla, César M. Flores-Ortíz, Fernando Candanedo, Miriam Rodriguez-Sosa, Sonia E. Hernández-Navia, Luis I. Terrazas

**Affiliations:** 1 Facultad de Estudios Superiores Iztacala, Unidad de Investigación en Biomedicina, Universidad Nacional Autónoma de México, Tlalnepantla, Edo. México 54090 México; 2 Facultad de Estudios Superiores Iztacala, Laboratorio Nacional en Salud, Universidad Nacional Autónoma de México, Tlalnepantla, Edo. México 54090 México; 3 Laboratorio de Fisiología Vegetal, Facultad de Estudios Superiores Iztacala, Unidad de Biología, Tecnología y Prototipos, Universidad Nacional Autónoma de México, Tlalnepantla, Estado de México 54090 México; 4 Departamento de Patología, Centro Médico Nacional SXXI, IMSS, Ciudad de México, México

**Keywords:** Colitis, Helminths, Carbohydrates, M2 macrophages, Glycans, Epithelial barrier, Adherens junction

## Abstract

**Background:**

Ulcerative colitis (UC) is a chronic inflammatory disease that is increasing in prevalence worldwide. Notably, helminth infections, known for their immunoregulatory properties, are inversely related to inflammatory conditions such as UC. Research has indicated that *Taenia crassiceps* infection can improve inflammatory-mediated diseases, including type 1 diabetes, experimental autoimmune encephalomyelitis, and colitis. Subsequent studies revealed that helminth-derived products can replicate the effects of complete infection in the context of inflammatory diseases; however, the mechanisms underlying these effects remain unclear. This study examined the impact of intact glycans from *T. crassiceps* excreted/secreted products (TcES) on host responses to dextran sodium sulfate (DSS)-induced colitis.

**Methods:**

UC was induced by administering 4% DSS in the drinking water for 9 days. The mice were treated with intact TcES, glycan-depleted TcES, or protein-depleted TcES 2 days after colitis induction. Symptoms of the disease, along with immunologic and histopathological evaluations, were performed.

**Results:**

Colitic mice that received intact TcES presented fewer disease symptoms and less histopathological damage. Intact TcES reduced the proinflammatory response while increasing the production of IL-4, IL-22, IL-31, and MCP-1 and promoting M2 macrophage polarization via PD-L2 expression. Furthermore, intact TcES diminished neutrophil infiltration, inhibited NF-κB and p38 phosphorylation in the colon, and suppressed reactive oxygen species and 3-nitrotyrosine levels, thus protecting the colon. These effects were accompanied by increased expression of E-cadherin and β-catenin, indicating improved epithelial barrier integrity. Conversely, mice treated with glycan-depleted or protein-depleted TcES exhibited exacerbated colitis characterized by disruption of colon tissue architecture, extensive inflammation, and epithelial damage, including loss of E-cadherin and β-catenin and a lack of M2 macrophage polarization.

**Conclusions:**

Glycoconjugates on TcES play a significant role in mediating the immunomodulatory effects that alleviate DSS-induced colitis.

**Supplementary Information:**

The online version contains supplementary material available at 10.1007/s10787-025-01821-y.

## Introduction

Ulcerative colitis (UC) and Crohn’s disease (CD) are inflammatory bowel diseases (IBD) characterized by a dysregulated immune response within the gastrointestinal tract mucosa that is influenced by complex interactions among environmental factors, the gut microbiome, and genetic predispositions (Kobayashi et al. 2020). UC is characterized by relapsing and remitting mucosal inflammation, typically initiating distally and potentially extending proximally to involve the entire colon (Nakase et al. 2021; Ramos and Papadakis 2019). The onset of UC commonly occurs in two distinct age groups: 20–30 years and 50–80 years (Nakase et al. 2021). Over the past half-century, the incidence of UC has steadily increased, particularly in North America, Europe, and Oceania. Projections estimate that the prevalence of IBD will reach 1% in the Western world by 2030 (Herauf et al. 2024; Kaplan and Windsor 2021). Standard treatments for UC include corticosteroids, 5-aminosalicylates, immune therapy, and immunosuppressive agents (Ananthakrishnan et al. 2024; Liu et al. 2022; Segal et al. 2021). However, a significant proportion of patients exhibit suboptimal responses to these conventional therapies, and the adverse effects associated with corticosteroids warrant the exploration of alternative therapeutic strategies for UC. In addition, UC patients face a 2–3-fold increased risk of developing colitis-associated colorectal cancer (CAC) due to chronic mucosal inflammation (Axelrad et al. 2022; Lutgens et al. 2013; Shah and Itzkowitz 2022). Interestingly, this chronic inflammatory state may be modulated by anti-inflammatory immune responses, such as those elicited by helminth infections and helminth-derived products, which have been shown to protect against IBD, positioning them as potential candidates for novel UC treatments (Alghanmi et al. 2024; Fawzy et al. 2024; Hou et al. 2022; Mighani et al. 2024; Rawat et al. 2024).

Helminth parasites have evolved sophisticated mechanisms to ensure their long-term survival within hosts, often inducing Th2-biased responses and altering innate immune cells such as macrophages and dendritic cells (Acevedo et al. 2024; Alghanmi et al. 2024). This immunomodulatory capacity, particularly the suppression of inflammatory responses, has prompted investigations into the therapeutic potential of helminths and their products in certain inflammatory-mediated diseases (Xie et al. 2024; Yang et al. 2024). *Taenia crassiceps,* a paratenic cestode parasite affecting rodents in its larval form, has demonstrated significant immunomodulatory effects in experimental models of autoimmune and inflammatory diseases, including colon cancer (Espinoza-Jiménez et al. 2010; León-Cabrera et al. 2014; Reyes et al. 2011). Infection with *T. crassiceps* induces the generation of alternatively activated macrophages, also known as M2 macrophages, which have suppressive functions and express IL-10, markers associated with tissue repair (arginase-1, Ym-1, Fizz-1), and immune checkpoints (PD-L1 and PD-L2) (Filbey et al. 2019; Prieto-Lafuente et al. 2009; Terrazas et al. 2005). Adoptive transfer of these M2 macrophages alleviated murine UC (Ledesma-Soto et al. 2015). Furthermore, *T. crassiceps* excreted/secreted products (TcES) exhibit anti-inflammatory properties by inhibiting dendritic cell maturation and pro-inflammatory cytokine production, such as IL-12p40, IL-12p70, IL-15, and TNF-α in response to LPS stimulation (Terrazas et al. 2010). TcES also reduced the production of inflammatory cytokines (IL-6, IL-12, and TNF-α) in bone marrow-derived macrophages stimulated with LPS while increasing the production of IL-10 and specific microRNAs, such as miR-125a-5p, miR-762, and miR-484 (Martínez-Saucedo et al. 2019) highlighting the potential of these molecules as therapeutic candidates for inflammatory diseases, such as UC. Conversely, proinflammatory responses and M1 macrophages have been implicated in the development of colitis (Jin et al. 2021; Wang et al. 2024, 2019, 2020; Xiao et al. 2024).

Helminths and their products include glycoproteins, glycolipids, and polysaccharides with *N-* and *O-*glycan structures (Kato and Heimburg-Molinaro 2024). *Schistosoma mansoni* egg glycans interact with C-type lectin receptors, demonstrating that these glycans mediate the interaction and recognition of helminths by the immune system (Meyer et al. 2007; Van Liempt et al. 2007). Glycans in the metacestode of *T. crassiceps* share structural similarities with the N-glycans of *S. mansoni* and induce IL-6 secretion via TLR-4 signaling in macrophages (Dissanayake et al. 2004). Moreover, carbohydrate-depleted TcES lose their ability to modulate TLR-mediated dendritic cell activation, supporting the importance of glycans in interactions with innate immune cells (Terrazas et al. 2010). Glycans on helminth-derived molecules have been recognized as critical in biasing Th2-type responses (Okano et al. 1999). However, the extent to which these glycans contribute to modulating inflammatory responses in vivo remains unclear. In this study, we aimed to investigate whether TcES can alleviate the pathologic symptoms of colitis and whether the glycans within TcES contribute to its potential protective effects.

## Materials and methods

### Animals

Eight- to nine-week-old female BALB/c mice were purchased from Harlan. All the mice were maintained in a free pathogen environment in the animal facility at Facultad de Estudios Superiores Iztacala, UNAM, where they received food and sterile water *ad libitum*. All animal studies were conducted in accordance with the guidelines of the Faculty Animal Ethical Care and Use Committee, as approved in reference CE/FESI/092017/1206, and the official Mexican regulation NOM-062-Z00-1999.

### Obtaining soluble products excreted/secreted by *Taenia crassiceps* (TcES)

Metacestodes of *T. crassiceps* (ORF strain) were obtained under sterile conditions from the peritoneal cavity of female BALB/c mice after 8 weeks of infection. The cysticerci were washed four times with sterile saline (0.9% NaCl) and cultured at 37 °C with 5% CO_2_ for 48 h. TcES was recovered from the supernatant and centrifuged for 10 min at 1000 × g. The supernatant was concentrated with a 50 kDa Amicon Ultra Filter (Millipore, Billerica, MA, US). A protease inhibitor (Sigma Aldrich, St. Louis, MO, US) was added, and the samples were stored at – 70 °C. The protein concentration and integrity were verified via the Bradford method (Sigma Aldrich, St. Louis, MO, US) and sodium dodecyl sulfate‒polyacrylamide gel electrophoresis (SDS‒PAGE) with Coomassie blue staining, respectively. The endotoxin level was evaluated via an E-TOXATE kit (Sigma‒Aldrich, St. Louis, MO, USA). According to this kit, TcES tested negative for endotoxin. Limit of sensitivity: 0.05–0.1 endotoxin units (EU) per ml.

### Depletion of carbohydrates by periodate treatment of TcES

Sodium metaperiodate-mediated modification of glycan molecules in TcES. TcES, 2 mg/mL, was incubated with 50-mM sodium acetate (pH 4.5) for 15 seconds at room temperature. The content was divided to produce periodate-modified extracts (TcES wo/c) and control mock treatment extracts (TcESm); these extracts contained all the salts except sodium metaperiodate. Two volumes of 20-mM sodium metaperiodate (Sigma‒Aldrich, St. Louis, MO, USA) were subsequently added. To the tube with the mock control, only 50-mM sodium acetate was added. Both tubes were incubated for 30 min in the dark at room temperature with gentle shaking. Subsequently, 100-mM sodium borohydride (Sigma Aldrich, St. Louis, MO, US) in PBS was added (vol/vol) to both tubes for 30 min at room temperature. Finally, the excess salt was removed via the dialysis of all the extracts and concentrated via 50 kDa Amicon Ultra Filter columns (Millipore, Billerica, MA, USA). The protein concentration was determined via the Bradford method, and protein integrity was assessed via SDS‒PAGE.

### Removal of proteins in TcES

To remove proteins from the TcES, 2 mg/ml of TcES was exposed to Proteinase K (Invitrogen, Thermo Fisher Scientific, Waltham, MA, USA) at a concentration of 200 μg/ml. The mixture was subsequently incubated at 59 °C for 4 h, and the proteinase K was inactivated at 80 °C for 1 h. SDS‒PAGE and Coomassie staining were used to verify protein integrity.

### Induction of colitis and treatments

BALB/c female mice received 4% dextran sulfate sodium (DSS) (MW: 35000–50000; MP Biomedical, Solon, OH, USA) in their drinking water for nine days to induce colitis. Five groups of mice were used (6 mice per group), but some of them died from the disease. However, all the experiments were repeated 2–3 times: control (water+200 μl of saline solution), DSS treatment + saline (4% DSS), and DSS + TcES at varying doses (4% DSS + TcES at 200 µg, 100 µg, and 50 µg daily, respectively). Dexamethasone-treated mice/groups (1 mg/kg and 0.5 mg/kg) served as controls and were administered intraperitoneally (Wang et al. 2017). In other experiments, seven groups of mice were induced: control (water + 200 μl saline solution), DSS-treated (4% DSS+200 μl saline solution), DSS + TcES (4% DSS + TcES 200 µg daily), TcES (water + TcES 200 µg daily), DSS+TcES wo/p (4% DSS+TcES 200 µg, proteins removed), DSS+TcES wo/c (4% DSS+TcES, carbohydrate depleted), and DSS+Mock (4% DSS+Mock, TcES containing all salts except sodium metaperiodate). TcES was administered intraperitoneally; all treatments began on day 2 after colitis induction. The mice were weighed daily to assess the percentage of body weight loss. To evaluate the disease activity index (DAI), body weight loss was scored from 0 to 8 (0–1, 0–1%; 2–3, 1–5%; 4–5, 6–10%; 6–7, 11–20%; 8, >20%), stool consistency was scored from 0 to 8 (0–1, normal; 2–3, soft, mucus stools; 4–5, loose stools; 6–7, diarrhea; 8, watery diarrhea), and rectal bleeding was scored from 0 to 7 (0–1, normal; 2–3, slight bleeding; 4–5, moderate bleeding; 6–7, gross bleeding.

### Alanine aminotransferase detection

To evaluate liver damage, serum samples from various groups of mice treated with dexamethasone and TcES were analyzed via a GPT alanine aminotransferase (ALT) kit (Spinreact, Sant Esteve de Bas, Girona, Spain). Twenty-five microliters of serum from each treatment group was measured according to the supplier's directions. The plate was read at 340 nm using a BioTek Epoch Microplate Spectrophotometer (Agilent, Santa Clara, CA, USA).

### Histology

Colon and liver tissue samples were collected, fixed in 100% ethanol, embedded in paraffin, sectioned at 4 µm, and stained with hematoxylin and eosin (H&E) to evaluate pathologic damage. Alternatively, the colon samples were stained with alcian blue to count the goblet cells in the colon. To assess the severity of DSS-induced colitis, histologic scores were determined on the basis of three independent parameters: extent, inflammation, and crypt damage. These factors were assessed and scored as follows: extent, scored from 0 to 3 (0, none; 1, mucosa; 2, submucosa; 3, muscular); inflammation, scored from 0 to 3 (0, none; 1, slight; 2, moderate; 3, severe); and crypt damage, scored from 0 to 4 (0, none; 1, basal one-third lost; 2, basal two-thirds lost; 3, only surface epithelium intact; and 4, entire crypt and epithelium lost). Goblet cell loss was evaluated by counting five fields (20X objective) per crypt in four crypts from each of five mice per group under different treatments. In the liver, the inflammatory infiltrate in the tissue was assessed by counting five fields in the portal triad and scoring them. The slides were analyzed via an AxioVert A.1 capture optical microscope (Carl Zeiss Microscopy GmbH).

### Flow cytometry

To determine the presence of M2 macrophages and myeloid-derived suppressor cells in the peritoneal cavity and colon, we used antibodies to characterize these populations from 1 × 10^6^ cells that had been washed with staining buffer (0.5% 1x-BSA and 0.5% sodium azide). The pellet was then Fcγ-RII blocked with CD16/CD32 for 20 min at 4 °C, washed, and subsequently stained with different antibodies: F4/80, MR, PD-L1, and PD-L2 (BioLegend, San Diego, CA, USA) for M2 macrophages and CD11b, Ly6C, and Ly6G (BioLegend, San Diego, CA, USA) for myeloid suppressor cells. The samples were incubated for 30 min at 4 °C and washed twice with staining buffer. Finally, the samples were resuspended in 500 µl of staining buffer and acquired via an Attune NxT flow cytometer (Thermo Fisher Scientific). The data were analyzed with FlowJo software (Tree Star, Inc., Ashland, USA).

### Immunoassay using multiplex MagPix

To identify different cytokines, we used the MAGPIX System (Luminex Corporation, USA) with a commercial kit, the Bio-Plex Pro Mouse Th17 Assay 10-Plex (Bio-Rad Laboratories, Hercules, CA, USA), according to the manufacturer’s protocol. The analytes were simultaneously detected in 25-µL samples of colon protein, and the mean fluorescence intensities were calculated via xPonent software.

### ELISA

Peripheral blood was collected and centrifuged at 2500 rpm for 10 min. The levels of TNF-α, IL-4, and MCP-1 in the serum of the mice were measured via a Mouse Enzyme-Linked Immunosorbent Assay Kit (Peprotech, Rocky Hill, NJ, USA) following the manufacturer’s instructions. The absorbance was measured at 405 nm via a Multiskan Ascent reader (Thermo Labsystems).

### TUNEL staining assay

Apoptosis of the colonic mucosa was detected via a terminal deoxynucleotidyl transferase dUTP nick-end labelling (TUNEL) in situ Cell Death Detection Fluorescein Kit (Roche, Risch-Rotkreuz, Switzerland) in paraffin sections (4 μm) according to the manufacturer's instructions. Cell apoptosis was observed via a Leica TCS SP8 confocal microscope and analyzed with ImageJ software. For the mean fluorescence, ten fields of each sample were analyzed.

### Reactive oxygen species (ROS) detection

The levels of reactive oxygen species (ROS) in the liver and colon protein extracts of the mice were determined through the reaction of the ROS with the compound 2′7′-dichlorofluorescein diacetate (DCFA-DA) (Sigma Aldrich, St. Louis, MO, USA). Liver and colon extracts were obtained via mechanical disruption. The oxidizing species interact with DCFA-DA, resulting in fluorescence that serves as an index of total ROS production in the sample. From each cell extract, 50 µl was placed in triplicate in a 96-well plate, and 195 µl of 1X PBS plus 5 µl of 500 µM DCFA-DA was added. The plate was placed in a fluorometer equipped with a 96-well plate reader (BioTek Instruments, Inc., Winooski, VT, USA), and fluorescence was recorded at an excitation wavelength of 485 nm and an emission wavelength of 520 nm for 60 min. The fluorescence value was reported as the relative fluorescence unit per mg of total protein in the crude extract.

### 3-nitrotyrosine detection

3-Nitrotyrosine production was evaluated in protein extracts from colon tissue across all treatments via ELISA following the manufacturer’s instructions (Abcam, Cambridge, CB2 OAX, UK). The antibody was coated onto a 96-well plate. Next, 50 μL of the samples (600 ng) and standards were added and incubated at room temperature for 2 h. The wells were washed again, and 50 μL of the detection antibody, anti-3-nitrotyrosine, was added. The mixture was then incubated for 1 h at room temperature. Afterward, the wells were washed, and HRP substrate solution (TMB) was added. Finally, the plaque was measured at 600 nm.

### Polymerase chain reaction

Total RNA was extracted via TRIzol reagent (Invitrogen, Carlsbad, CA, USA) according to the manufacturer´s instructions. Then, we performed inverse transcription via the RevertAid H minus First Strand cDNA Synthesis Kit (Thermo Scientific, Rockford, IL, USA). The obtained complementary DNA was amplified via polymerase chain reaction (Amplificase; BioTecMol, México City, México) via the following primer set: mouse GAPDH (forward), CTC ATG ACC ACA GTC CAT GC; mouse GAPDH (reverse), CAC ATT GGG GGT AGG AAC AC; mouse Arg-1 (forward), CAG AAG AAT GGA AGA GTC AG; mouse Arg-1 (reverse), CAG ATA TGC AGG GAG TCA CC; mouse Ym-1 (forward), TCA CAG GTC TGG CAA TTC TTC TG; Ym-1 (reverse), TTT GTC CTT AGG GCT TCC TC; mouse TNF-α (forward), GGC AGG TCT ACT TTG GAG TCA TTG C; mouse TNF-α (reverse), ACA TTC GAG GCT CCA GTG AAT TCG; mouse IL-10 (forward), ACC TGG TAG AAG TGA TGC CCC AGG CA; mouse IL-10 (reverse), CTA TGC AGT TGA TGA AGA TGT CAA A. The images were taken on the Gel Doc EZ Imager (Bio-Rad Laboratories, Hercules, CA, USA). The relative expression levels of the target genes were normalized to those of the GAPDH gene.

### Immunofluorescence

Distal colon tissues were embedded in paraffin, and four-micron-thick colon sections were deparaffinized and boiled with an antigen retrieval DIVA Decloaker (Biocare Medical; CA, USA). After the tissue was blocked with PBS-BSA 1% for 1 h at room temperature, the primary antibodies were diluted with PBS-BSA 1% for Arginase-1 (1:50) and iNOS (1:100) (Cell Signaling Technology, MA, USA) and incubated overnight at 4 °C. After the addition of the secondary antibody anti-rabbit-FITC (1:200) (Invitrogen, USA) and incubation for 1 h at room temperature, the nuclei were stained with mountain medium with DAPI (Abcam; MA, USA). Images were captured via a Leica TCS SP8X confocal microscope.

### Immunohistochemistry

The slides were deparaffinized and subjected to antigen retrieval, which was carried out by incubation with a DIVA Decloaker (Biocare Medical; CA, USA). After heating, endogenous peroxidase activity was inhibited with 3% hydrogen peroxide in methanol for 10 min, and the slides were washed and blocked with 1 × BSA 1% in PBS for 1 h. Primary antibodies against mouse E-cadherin (1:300), β-catenin (1:500), and active β-catenin (1:100) (Cell Signalling; MA, USA) were incubated with the samples at 4 °C overnight. Next, the slides were washed and incubated with an HRP-conjugated secondary anti-rabbit antibody (1:500) (BioLegend; CA, USA) for 1 h at room temperature. Finally, the sections were stained with a diaminobenzidine (DAB) chromogen kit (Abcam; MA, USA) for 5 min and counterstained with hematoxylin. The mean density of the positive area was analyzed by the image ratio.

Colon tissues were cut and frozen in liquid nitrogen, pulverized, and placed in lysis buffer via the RNA/DNA/Protein Purification Plus Kit (Norgen Biotek: Ontario, Canada) as specified by the manufacturer. Protein quantification was performed with a bicinchoninic acid (BCA) kit (Thermo Scientific, Rockford, IL, USA). For 30 μg of protein, separation was performed via SDS‒PAGE in a Mini-PROTEAN Tetra cell (Bio-Rad; Mexico) for 2 h at 90 V. Subsequently, the proteins were transferred to a PVDF membrane (Millipore, Billerica, MA, US) for 1.5 h at 80 V at 4 °C. The membrane was then blocked with 5% nonfat milk for 1 h at room temperature. Afterward, the membranes were incubated overnight at 4 °C with the primary antibodies anti-β-actin (BioLegend; CA, USA), diluted 1:2000, and anti-NF-κB (p65) total and anti-P38 total and phosphorylated (Cell Signalling; MA, USA), diluted 1:1000. The membranes were then washed five times for 10 min each with TBS-Tween. The membranes were subsequently treated with HRP-conjugated donkey anti-rabbit IgG (BioLegend; CA, USA) diluted 1:5000 and incubated for 2 h at room temperature after being washed five times with TBS-Tween. Finally, the membranes were visualized via chemiluminescence via SuperSignal West Femto Maximum Sensitivity Substrate (Thermo Scientific; Rockford, IL, USA) in a C-DiGit Blot Scanner (LI-COR Bioscience). The images were quantified via ImageStudio 4.0 software (LI-COR Bioscience) and normalized to β-actin.

### Lectin blot with concanavalin A and wheat germ agglutinin

To perform lectin blotting with concanavalin A (ConA), 30 μg of proteins from intact TcES, TcES depleted of carbohydrates (TcES wo/c), and TcES mock were loaded onto a 10% SDS‒PAGE gel and run for 1 h and 30 min at 90 V. After that, the gels were placed in lectin buffer (0.1-M NaCl, 0.05-M Tris‒HCl, 1-mM CaCl_2_, 1-mM MnCl_2_), where the buffer was incubated at room temperature with shaking and replaced every 5 min. Next, ConA, coupled to FITC at a concentration of 1 mg/ml, was added to the gel and incubated overnight at 4 °C. The gels were subsequently washed three times with lectin buffer for 5 min each. Finally, the gels were visualized via an ultraviolet filter with a Gel Doc EZ Imager (Bio-Rad Laboratories, Hercules, CA, USA) at 366 nm. To conduct the lectin blot with wheat germ agglutinin (WGA), we used 10 μg of proteins from intact TcES, TcES depleted of carbohydrates (TcES w/o c), and TcES mock, which were loaded onto a 4–20% Bis-Tris SDS‒PAGE gel. The proteins were transferred to a PVDF membrane for 2 h at 4°C. The membranes were blocked with 5% nonfat milk for 1 h at room temperature and then incubated with HRP-lectin WGA (Sigma Aldrich, St. Louis, MO, USA) at a concentration of 1 μg/ml in 5% PBS-BSA for 2 h at room temperature. The membranes were washed with 0.04% PBS-Tween five times for 5 min. Chemiluminescence was developed via the SuperSignal West Femto Maximum Sensitivity Substrate (Thermo Scientific; Rockford, IL, USA), which was diluted 1:12. The glycosylated proteins were then detected and visualized via the Alliance Uvitec Cambridge system (Thermo Scientific; Rockford, IL, USA).

### Fingerprint of carbohydrates by infrared spectroscopy

The infrared (IR) spectra of various carbohydrates, including standard d-glucose, d-mannose, d-galactose, and d-fructose (Sigma Aldrich, St. Louis, MO, USA), were determined via IR spectroscopy at concentrations ranging from 0 to 20 mg/ml. A volume of 10 μL of each carbohydrate mixture was placed in a PerkinElmer Spectrum FT-IR/NIR spectrometer. The fingerprint spectrum was then obtained in the 1450–650 cm⁻^1^ range. Different *Taenia*-derived products were evaluated: intact TcES, TcES wo/c, TcES wo/p, and TcES mock. For TcES wo/p, the wavelength range was 1560–650 cm^2^, and the spectrum was detected via PerkinElmer Spectrum software version 10.53.738.

### Statistical analysis

The data were normalized via the Shapiro‒Wilk test; subsequently, they are expressed as the means ± standard deviations (SDs) from two or more independent experiments. The data were analyzed via an unpaired Student’s *t* test for comparisons between two groups and one-way ANOVA with Tukey’s test for comparisons among multiple groups. GraphPad Prism 8 software (GraphPad) was used for all the statistical analyses; *p* values less than 0.05 were considered statistically significant.

## Results

### TcES administration ameliorates DSS-induced colitis

**Fig. 1:**
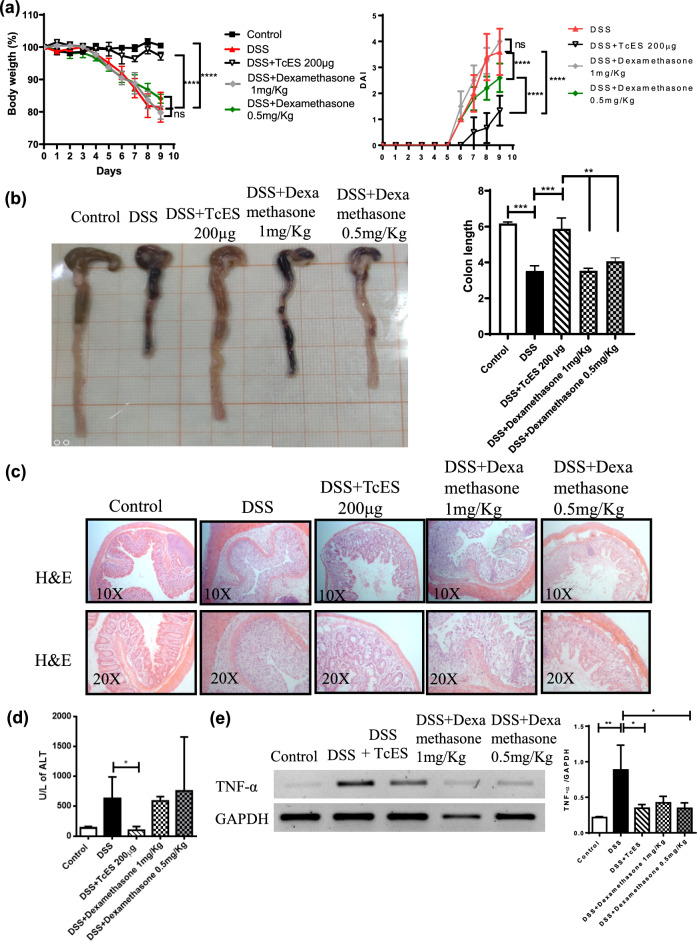
Comparison of the effects of TcES treatment and dexamethasone treatment in a DSS-induced colitis model. **a** Body weight loss (*n* = 4–6) and the disease activity index (DAI) were evaluated across all conditions for 9 days. **b** Changes in the macroscopic appearance of the colon and a graphic of colon length in response to different treatments (*n* = 4–6). **c** Histology of the colon under different conditions (*n* = 4–6), stained with H&E. **d** Representative images of H&E-stained liver tissue from the different treatment groups and a graphic of the inflammatory infiltrate in the liver from five fields near the portal triad (*n* = 4–6). **e** Serum ALT levels (*n* = 4–6). **f** RT‒PCR expression of the TNF-α gene in colon tissue and densitometry with GAPDH as a housekeeping gene. The data are shown as the mean ± SD, *p ≤ 0.05, **p ≤ 0.005. Statistical analysis was performed with one-way ANOVA followed by Tukey’s test

Research from our laboratory has indicated that preinfection with the helminth *Taenia crassiceps* modulates the progression of experimental colitis. Given the impracticality of employing live parasite infections as therapeutic interventions, even with species that are innocuous to humans, we aimed to determine whether the effects of this whole infection could be replicated by administering *T. crassiceps*-excreted/secreted molecules (TcES) to mitigate experimental colitis. Initially, a dose‒response assay (50, 100, and 200 μg/mouse) revealed that 200 μg/mouse of TcES provided the optimal response to modulate colitis (Supplemental Fig. 1a–c). To further assess the regulatory effect of TcES on DSS-induced colitis, we compared this TcES dose with dexamethasone at doses of 1 mg/kg and 0.5 mg/kg. Treatments commenced on day 2 after DSS administration (4% in drinking water) and continued for seven days. The mice were monitored daily for signs of disease. As anticipated, dexamethasone treatment attenuated some colitis symptoms, particularly at a dose of 0.5 mg/kg, including DAI, colon shortening, and histologic damage. Notably, TcES treatment elicited a more substantial and significant suppression of this pathology (Figure 1a–c). DSS-induced colitis resulted in elevated alanine aminotransferase (ALT) levels, which are indicative of hepatic alterations, and dexamethasone treatment fails to reverse these changes. Conversely, TcES treatment restored ALT levels to normal values (Fig. 1e). Furthermore, histopathological examination of the liver confirmed DSS-induced hepatic damage reflected by elevated ALT levels; TcES reduced DSS-induced hepatitis, suggesting the absence of hepatic toxicity associated with TcES administration (Fig. 1d). In contrast, dexamethasone treatment, despite reducing hepatitis, also caused adverse effects on the liver parenchyma, characterized by hepatocyte swelling and sinusoidal dilation (Fig. 1d). RT‒PCR analysis of the colon revealed that TcES and dexamethasone in both doses reduced TNF-α expression (Fig. 1f).

**Fig. 2:**
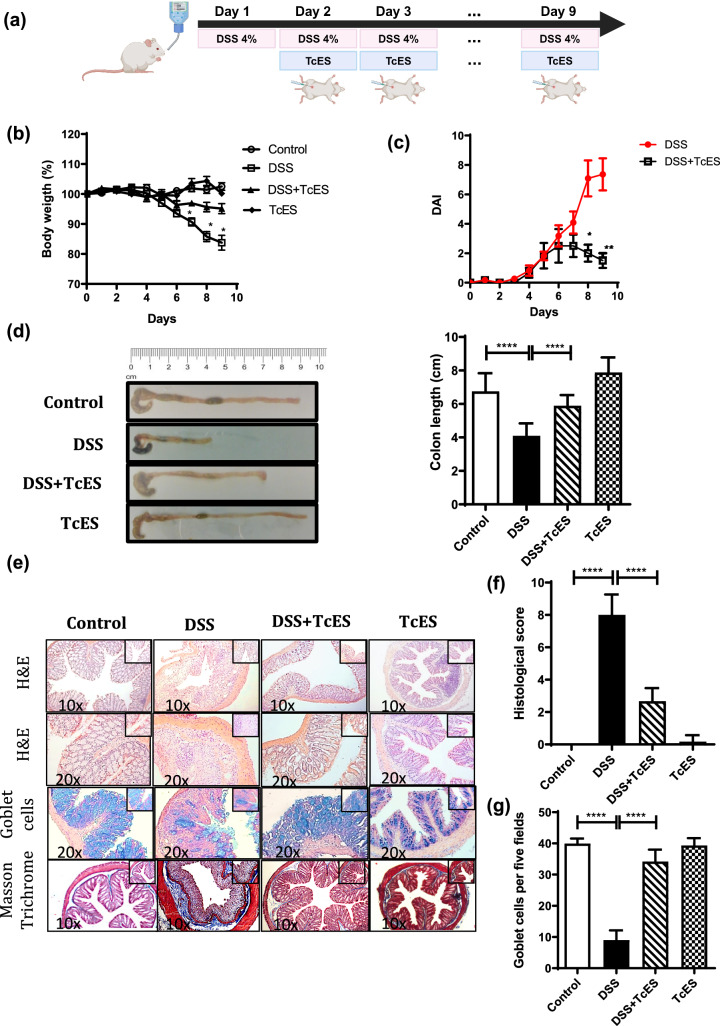
Effects of TcES treatment on the symptoms and pathology of DSS-induced colitis. **a** Study design, created with BioRender. **b** Daily body weight loss (*n* = 6). **c** The disease activity index (DAI) was evaluated on the basis of body weight loss, stool consistency, and rectal bleeding (*n* = 6). **d** Macroscopic appearance of the colon (left) and graphic of colon length measured from the proximal colon to the rectum (right) (*n* = 6). **e** TcES administration protected mice from DSS-induced colon damage. Colon histology was performed via H&E, Alcian blue, and Masson’s trichrome staining. **f** Histologic damage was evaluated by the infiltration of cells, disruption of epithelial cells, and loss of crypts in the tissue (*n* = 6). **g** Goblet cells were evaluated by counting five fields (20X objective) per crypt of four crypts per mouse under different conditions (*n* = 4–6). The experiments were independently repeated three times. Each bar represents the mean ± SEM of six mice per group. *p ≤ 0.01, ***p ≤ 0.001, ****p ≤ 0.0001. The test was one-way ANOVA followed by Tukey’s test and a *t* test

After establishing the efficacy of TcES (200 μg) over dexamethasone, a commonly used drug for treating inflammatory diseases, we further investigated its potential anticolitic effects. The mice received 4% DSS in their drinking water, followed by daily intraperitoneal injections of TcES (200 μg) starting on day 2 (Fig. 2a). We monitored the mice daily for signs of disease, including weight loss, DAI, colon length, histopathological damage, and the number of colonic goblet cells. After five days of DSS-induced colitis, untreated mice presented typical signs of colitis, such as weight loss, piloerection, and a relatively high DAI. By day 9, they had significant histological damage, including loss of crypts, disrupted epithelial cells, loss of goblet cells, high inflammatory infiltration in the mucosa, edema in the submucosa, and a significantly shortened colon, compared with those of healthy mice (Fig. 2b–f). In contrast, TcES treatment alleviated the symptoms and pathology of DSS-induced colitis, reducing weight loss and DAI scores (Fig. 2b, c). This treatment successfully reversed the expected degree of colon shortening (Fig. 2d). Histologic evaluation of their colons revealed reduced mucosal cell infiltration, fewer disrupted epithelial cells, and well-preserved crypt architecture (Fig. 2e, f). Similarly, compared with untreated DSS-induced colitis mice, DSS-induced colitis mice treated with TcES presented an increased number of goblet cells (Fig. 2g). Furthermore, this treatment did not induce fibrosis in the tissue, as evidenced by Masson’s trichrome staining (Fig. 2e). The mice that received TcES alone showed no adverse reactions to the treatment (Fig. 2b, d–g).

### TcES modulates cytokine production in the colon microenvironment of mice with colitis

**Fig. 3:**
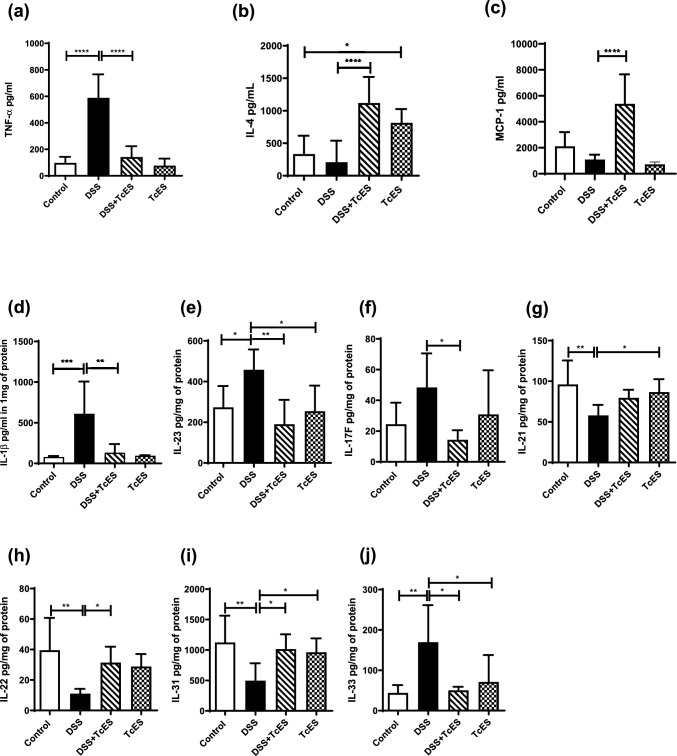
TcES modulates systemic and microenvironment cytokines in colon tissue. **a**–**c** Cytokines in the circulation were measured in the serum of the mice in the different groups via ELISA (*n* = 5). **d**‒**k** Cytokine levels in colon tissue protein extracts were detected via multiplex technology (*n* = 6). The data are shown as mean± SD, *p ≤ 0.05, **p ≤ 0.01, ****p ≤ 0.0001. Statistical analysis was performed with one-way ANOVA followed by Tukey’s test

To elucidate the potential of TcES treatment to modulate the proinflammatory microenvironment within the colons of DSS-induced colitis mice, we quantified the production of various cytokines associated with proinflammatory responses, such as TNF-α, IL-1β, IL-23, IL-17F, IL-21, and IL-33, as well as those involved in epithelial barrier protection, including IL-22, IL-31, and MCP-1, which are implicated in monocyte chemotaxis, and IL-4. Inflammatory cytokines play crucial roles in the development of ulcerative colitis, as shown in DSS-treated mice, which presented significantly increased levels of inflammatory cytokines, including IL-1β, IL-23, and IL-17F, in colon extracts, whereas TNF-α levels were elevated in the serum (Fig. 3a, d–f). Increased levels of IL-33 were also observed in the colon (Fig. 3j). Conversely, the colitic mice presented reduced production of IL-21, IL-22, and IL-31 (Fig. 3g–i, k). In contrast, DSS-induced colitis mice that received TcES exhibited significant decreases in the production of TNF-α, IL-1β, and IL-33 (Fig. 3a, d, j), as well as Th17-associated cytokines such as IL-23 and IL-17F (Fig. 3e, f). In addition, DSS-induced colitis mice treated with TcES showed a significant increase in the production of IL-4 (Fig. 3b) and the chemokine MCP-1 (CCL2), along with increased production of IL-22 and IL-31, both of which are cytokines involved in protecting the epithelial barrier (Fig. 3c, h, i).

### TcES promotes M2 macrophage polarization

**Fig. 4:**
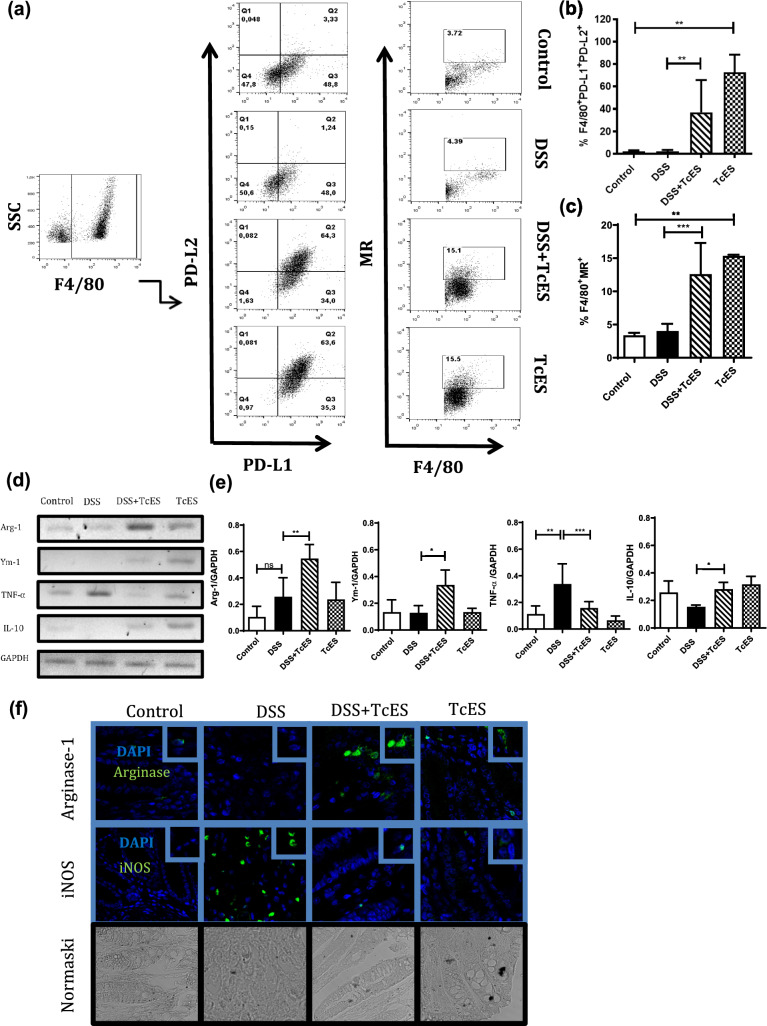
Detection of M2 macrophage polarization in vivo in the peritoneal cavity and colon. M2 macrophages in the peritoneal cavity were identified by the expression of F4/80, MMR, PD-L2, and PD-L1 in all groups and were analyzed by flow cytometry. A representative dot plot from the flow cytometry analysis is shown below. **b** F4/80, PD-L1, and PD-L2 (M2) frequencies of macrophages in the peritoneal cavity (*n* = 4–6). **c** Frequency of F4/80 and MMR (M2) macrophages in the peritoneal cavity (*n* = 4–6). **d** Expression of the genes associated with the M2 profile, Arginase-1, Ym-1, and IL-10, and the M1 profile, TNF-α, was assessed by PCR in the colon. GAPDH was used as a constitutive control. **e** The relative mRNA expression levels of Arginase-1, Ym-1, TNF-α, IL-10 and TNF-α were normalized to those of the GAPDH gene (*n* = 5). **f** Immunofluorescence detection of arginase-1 (M2), iNOS (M1), and DAPI in colon tissue from the different groups. The experiments were independently repeated three times. The data are presented as the means ± SDs of each group. *p ≤ 0.05, **p ≤ 0.01, ***p ≤ 0.001. *ns* not significant. A statistical test was performed via one-way ANOVA, followed by Tukey’s test

Several studies have suggested that M2 macrophages play a role in attenuating DSS-induced colitis. Therefore, we investigated whether TcES could induce an M2 macrophage profile in the peritoneum and colon. Flow cytometry analysis revealed that TcES significantly increased the percentage of F4/80+PDL1+PDL2+ (Fig. 4a, b) and F4/80+MR+ (Fig. 4c) peritoneal macrophages in both healthy and DSS-colitis mice. These surface markers are characteristic of M2-type macrophages and are consistent with the increased IL-4 production induced by TcES. Gene expression analysis of the colon (Fig. 4d) revealed that DSS increased TNF-α expression, whereas TcES significantly reduced TNF-α expression and increased Arg-1, Ym-1, and IL-10 expression (Fig. 4e). Finally, immunofluorescence analysis of iNOS and arginase-1 protein expression in the colon (Fig. 4f) confirmed that DSS-induced colitis promoted iNOS expression, which was reduced by TcES treatment, while arginase-1 expression increased (Fig. 4f).

### TcES decreases neutrophil infiltration, reactive oxygen species (ROS) production, 3-nitrotyrosine formation, and apoptosis in mice with colitis

**Fig. 5:**
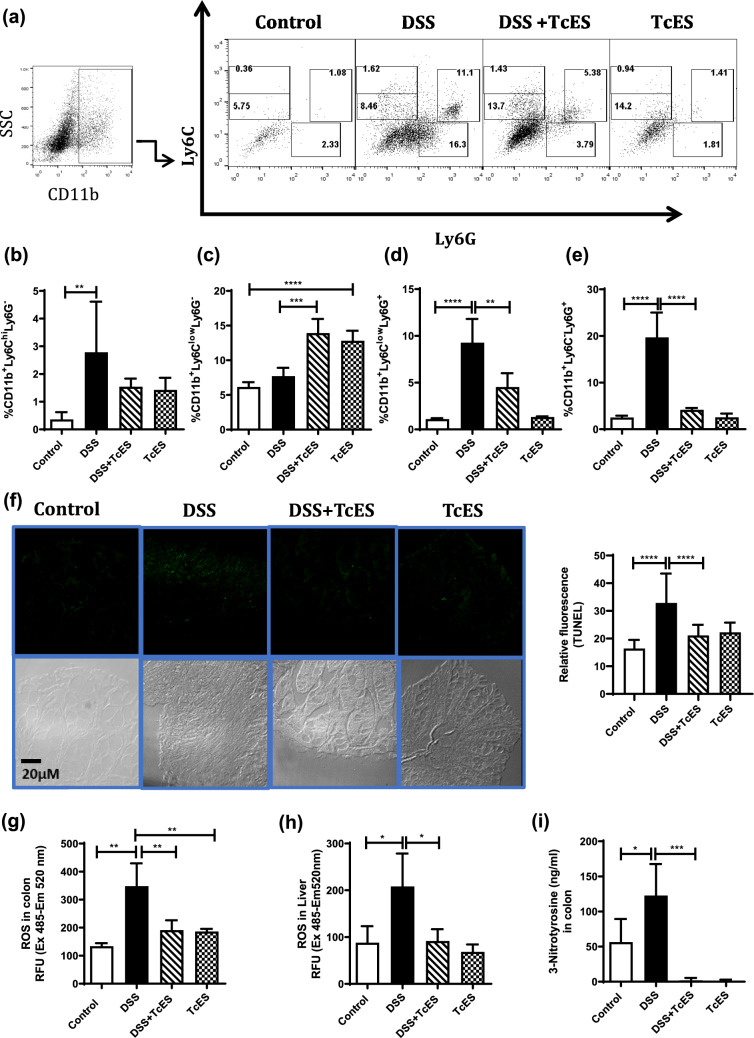
Evaluation of neutrophil infiltration, apoptosis, ROS, and 3-nitrotyrosine production in colitis model mice. **a** Dot plot of lamina propria cells showing the expression of CD11b, Ly6C, and Ly6G determined by flow cytometry (*n* = 5). **b** Frequency of the expression of the inflammatory monocytes CD11b^+^Ly6C^hi^Ly6G^-^. **c** Frequency of CD11b^+^Ly6C^low^Ly6G^-^ cells. **d** CD11b^+^Ly6C^low^Ly6G^+^ cells. **e** CD11b^+^Ly6C^-^Ly6G^+^ cells in the colon (*n* = 5). **f** Apoptosis detection by TUNEL in colon tissue; scale bar, 20 µm. The relative fluorescence in the colon was used to determine apoptosis (*n* = 5). **g** Extracts of colon tissue protein were used to determine the ROS levels induced by Diclorofluorescein (*n* = 4–5). **h** Detection of ROS in liver protein extracts from different conditions (*n* = 4–6). **i** Production of 3-nitrotyrosine in colon protein extracts from different treatment groups (*n* = 4–6). The experiments were repeated three times independently. The data are shown as the means± SDs; *p ≤ 0.05, **p ≤ 0.01, ****p ≤ 0.0001. *ns* not significant. Statistical analysis was performed with one-way ANOVA followed by Tukey’s test

Tissue-resident macrophages in the intestine are constantly replenished with monocytes attracted by local inflammatory stimuli (Bain et al. 2014; Ma et al. 2022; Mouhadeb et al. 2018). On the basis of the observed increase in MCP-1 production following TcES treatment, we investigated monocyte and granulocyte infiltration into the colon. Flow cytometry was performed on infiltrated cells from the lamina propria to detect CD11b^+^Ly6C^hi^Ly6G^-^, CD11b^+^Ly6C^low^Ly6G^-^, CD11b^+^Ly6C^low^Ly6G^+^, and CD11b^+^Ly6C^-^Ly6G^+^ (granulocytes) cell populations. As shown in the dot plot in Fig. 5a, DSS-induced colitis increased the infiltration of monocytes and neutrophils to the lamina propria CD11b^+^Ly6C^hi^Ly6G^-^ (Fig. 5b) and CD11b^+^Ly6C^low^Ly6G^+^ (Fig. 5d) and CD11b^+^Ly6C^-^Ly6G^+^ (Fig. 5e) populations. In contrast, TcES treatment significantly reduced neutrophil infiltration into the colon (Fig. 5d, e) while increasing the percentage of CD11b^+^Ly6C^low^Ly6G^-^ monocytes recruited to the colon (Fig. 5c). According to our TUNEL assays, compared with healthy mice, DSS-induced colitis mice showed a significant increase in apoptotic cells compared to the healthy group (Fig. 5f); however, TcES reduced the number of apoptotic cells in the colons of DSS-induced colitis mice (Fig. 5f).

Inflammation in individuals with colitis generates high levels of reactive oxygen species (ROS) in both the colon and the liver. Protein extracts from these tissues were mixed with dichlorofluorescein diacetate (DCFA-DA) to assess the levels of ROS produced by colitis in the different groups. Our results revealed that mice with colitis exhibited increased ROS production in the colon and liver (Fig. 5g, h), whereas treatment with TcES reversed this production in both tissues (Fig. 5g, h). Furthermore, we analyzed the generation of 3-nitrotyrosine (3-NT), a biomarker of oxidative stress and protein damage, in the colon. We found that colitic mice significantly increased the formation of 3-NT, whereas TcES markedly inhibited its generation.

### TcES attenuates nuclear factor-kappa B (NF-κB) and p38 phosphorylation in DSS-induced colitis

**Fig. 6:**
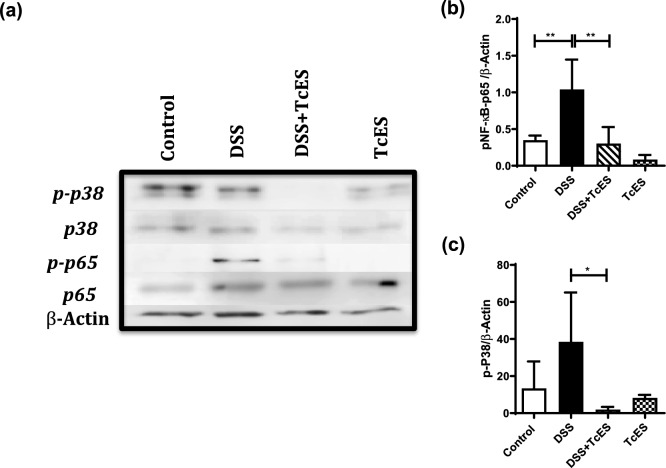
Phosphorylation of NF-κB and p38 in the colons of DSS-induced colitis mice. **a** Western blot showing the protein levels of p38, p-p38, NF-κB p65, NF-κB p-p65, and β-actin in colonic homogenates. **b, c** The bars representing the phosphorylation levels of NF-κB p-p65 and p-p38 in colon tissue were normalized to that of β-actin (*n* = 4–6). The experiments were repeated three times independently. Each bar represents the mean ± SEM of four mice per group. *p ≤ 0.05, **p ≤ 0.005. The test was one-way ANOVA, followed by Tukey’s test

The NF-κB pathway is inherently linked to the signaling and transcription of many inflammatory cytokines, whereas the p38 pathway is associated primarily with the induction of apoptosis. To elucidate the potential impact of TcES treatment on the NF-κB and p38 signaling pathways, Western blot assays were conducted to evaluate the phosphorylation status of these signaling molecules in colon tissue (Fig. 6a). Following DSS-induced colitis, a significant increase in the phosphorylation levels of both NFκB (p65) and p38 was observed in colon tissue (Fig. 6b, c). In contrast, TcES treatment significantly reduced the phosphorylation of both p65 and p38 (Fig. 6b, c).

### Glycans within TcES are critical for protection against DSS-induced colitis

**Fig. 7:**
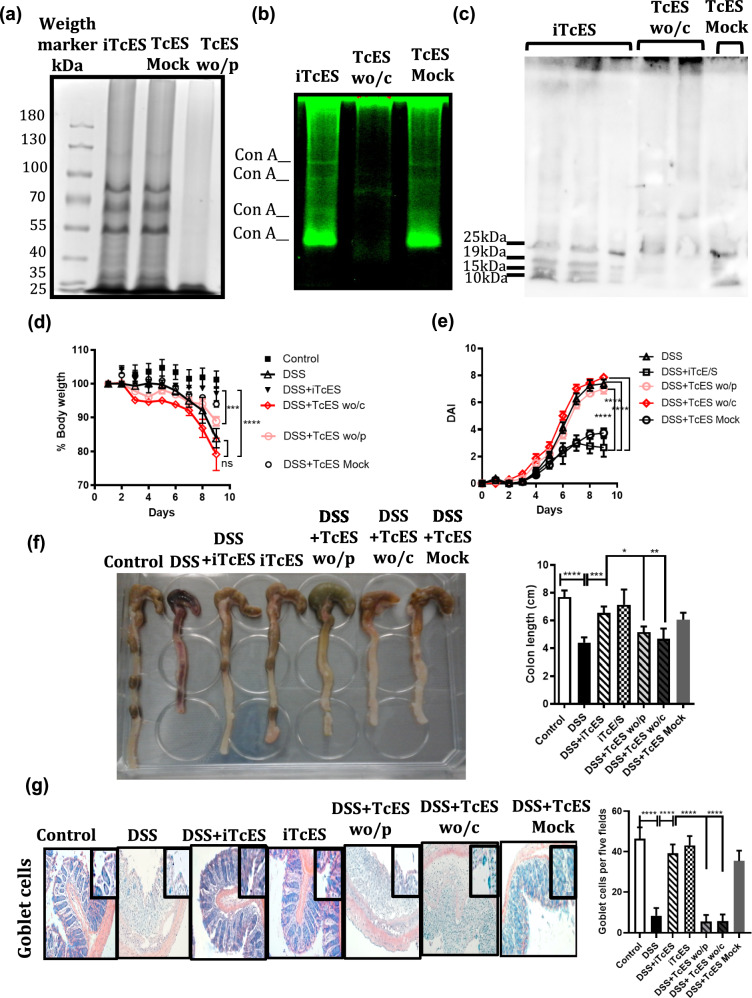
Protein integrity and lectin blot analysis of TcES and its effect on colitis. **a** SDS‒PAGE gel showing the proteins in intact TcES and protein degradation in TcES by proteinase K. **b** Lectin blot with concanavalin A FITC-conjugated (ConA) gel was used to determine the presence of carbohydrates in TcES. **c** Lectin blot of intact TcES, TcES wo/carbohydrates, and TcES mock in a gradient 4–20% Bis-Tris SDS‒PAGE gel, transferred to PVDF and incubated with HRP-lectin WGA. **d** Weight loss was determined daily (*n* = 5). **e** The DAI was evaluated via the parameters of body weight loss, stool consistency, and rectal bleeding (*n* = 5). **f** Macroscopic appearance of the colon (left) and graphic of the colon length measured from the proximal colon to the rectum (right) (*n* = 5). **g** Goblet cells were evaluated under different conditions via alcian blue staining, and five fields per mouse tissue sample were counted (*n* = 5). Each experiment was repeated three times. The data are presented as the means ± SDs of each group. *p ≤ 0.05, **p ≤ 0.01, ***p ≤ 0.001, ****p ≤ 0.0001. A statistical test was performed via one-way ANOVA followed by Tukey´s test and two-way ANOVA followed by Turkey´s test.

To investigate and delineate the respective roles of carbohydrates and proteins from TcES in mitigating colitis progression, we utilized sodium metaperiodate oxidation and proteinase K digestion to selectively deplete carbohydrates (glycans) and proteins from TcES, respectively. The effective removal of proteins from TcES was verified via 7% SDS‒PAGE, which compared intact TcES (iTcES) with mock TcES, which was used as a control and lacked sodium metaperiodate treatment (Fig. 7a). To validate glycan depletion in TcES samples, a lectin blot assay was performed using the ConA FITC conjugate, and the results were compared with those of iTcES and the TcES mock. This analysis revealed several ConA-recognized glycoproteins in TcES (Fig. 7b), which is consistent with prior research. Furthermore, we identified the presence of β (1-4) N-acetyl-d-glucosamine within TcES via WGA lectin. We found that iTcES contained these glycans, which were primarily localized in low-molecular-weight proteins (Fig. 7c). The presence of d-glucose, d-mannose, d-galactose, and d-fructose carbohydrates, recognized by ConA and WGA lectins in TcES, was further substantiated through infrared spectroscopy, analyzing the fingerprint region (1450–650 cm^−1^) and the concentration of different carbohydrates in the composition of TcES. The infrared spectra indicated the presence of d-glucose, d-mannose, and d-galactose in iTcES (Supplementary Fig. 2a–c), as evidenced by the spectral resemblance between each carbohydrate and iTcES. However, the slight shifts observed in the iTcES peaks suggest potential coupling of these carbohydrates to proteins or lipids. Notably, the d-fructose fingerprint did not align with the iTcES fingerprint (Supplementary Fig. 2d). The absence of glycans in the TcES wo/c samples was also confirmed (Supplementary Fig. 2a–d). Conversely, the presence of carbohydrates was detected in TcES wo/p (Supplementary Fig. 2e–h). Quantification of the carbohydrate composition of iTcES, achieved through spectral deconvolution of infrared spectra, revealed that d-galactose (24.33 μg/ml) was the most abundant carbohydrate, followed by d-glucose (11.49 μg/ml) and d-mannose (11.12 μg/ml) (Supplementary Fig. 2i).

To evaluate whether the potential ability of iTcES to alleviate DSS-induced colitis is attributable to its glycan content, mice with DSS-induced colitis were treated with intact TcES (iTcES), TcES without carbohydrates (TcES wo/c), TcES without proteins (TcES wo/p), or TcES mock. The absence of carbohydrates or proteins in TcES did not protect against DSS-induced colonic damage, leading to significant and rapid weight loss similar to that observed in the DSS-only group (Fig. 7d), accompanied by elevated DAI scores (Fig. 7e) and reduced colon lengths (Fig. 7f). Furthermore, a histologic examination of colon tissue revealed that the depletion of carbohydrates or proteins from TcES exacerbated mucosal damage, characterized by epithelial cell destruction and crypt distortion. A decrease in the number of goblet cells, indicative of compromised protective function, was also noted in the absence of carbohydrates or proteins in TcES (Fig. 7g), in contrast to the results of the mock treatment, in which no tissue damage or reduction in the number of goblet cells was evident.

### Glycans within TcES protect the epithelial barrier

**Fig. 8:**
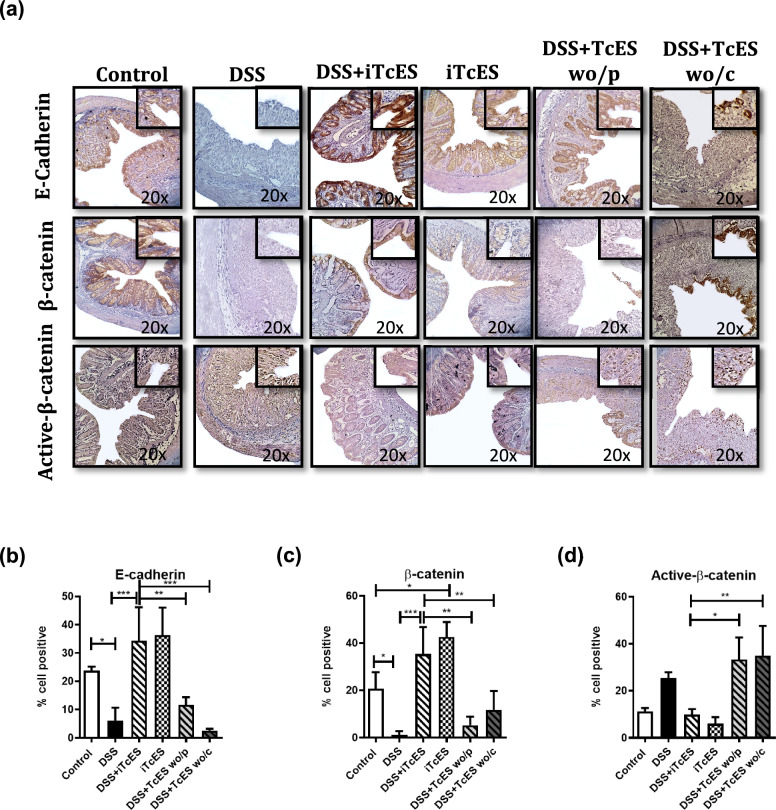
Colon expression of E-cadherin, β-catenin, and active β-catenin in the DSS-induced colitis model. **a** Immunohistochemical detection of E-cadherin, β-catenin, and active β-catenin in the colon. **b**–**d** The percentages of cells positive for E-cadherin, β-catenin, and active β-catenin were determined via the ImageJ software (*n* = 5). Each experiment was repeated three times. The data are presented as the means ± SDs of each group. *p ≤ 0.05, **p ≤ 0.01, ***p ≤ 0.001, ****p ≤ 0.0001. A statistical test was performed via one-way ANOVA, followed by Tukey’s test

The maintenance of microbiota–colon tissue homeostasis relies on an intact epithelial barrier, which comprises tight junctions, desmosomes, and adherens junctions. E-cadherin and catenin family members constitute the core components of adherens junctions (Garcia et al. 2018). Epithelial barrier dysfunction is a hallmark of colitis development (Nighot et al. 2023; Mansouri et al. 2024; Mehandru and Colombel 2021). To investigate whether TcES may protect the colonic epithelial barrier and to determine whether this protection is mediated by its glycan composition, we performed immunohistochemical staining to detect E-cadherin, β-catenin, and active β-catenin across all experimental groups. Our findings indicated that under DSS-induced inflammatory conditions, E-cadherin and β-catenin expression were significantly reduced by 75% and 94%, respectively (Fig. 8a–c), whereas active nuclear β-catenin expression was increased by 1.48-fold (Fig. 8d). In contrast, the colitic mice treated with iTcES displayed a significant increase in E-cadherin and β-catenin expression in colonic epithelial cells (Fig. 8b, c) and a 46% reduction in active nuclear β-catenin (Fig. 8d). Furthermore, iTcES alone significantly increased cytoplasmic β-catenin expression. However, the absence of glycans in TcES attenuated the expression of E-cadherin and β-catenin (Fig. 8b, c) while augmenting active β-catenin expression (Fig. 8d) and failed to reverse colonic tissue damage (Fig. 8a).

### Glycans in TcES promote M2 macrophage polarization in the peritoneum and colon

**Fig. 9:**
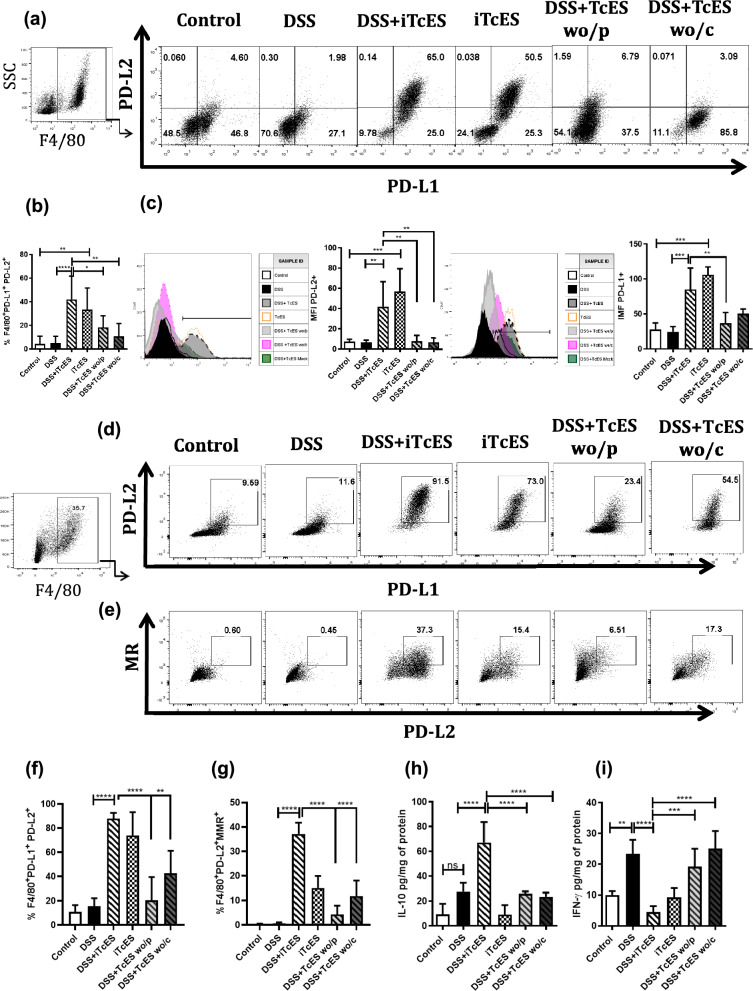
Analysis of M2 macrophages in the peritoneal cavity and colon. Cells from the peritoneal cavity and colon lamina propria were stained for F4/80, PD-L1, PD-L2, and MR, and flow cytometry was performed in DSS-induced colitis mice. **a** Dot plot of F4/80, PD-L1, and PD-L2 expression. **b** F4/80, PD-L1, and PD-L2 frequencies in peritoneal cavity cells (*n* = 4‒6). **c** Histogram of the expression of PD-L2 and PD-L1 and their median fluorescence intensity (MFI) values (*n* = 4–6). **d** Dot plot of macrophages in the colon evaluated through F4/80, PD-L1, and PD-L2 expression. **e** Dot plot of F4/80, PD-L2, and MR cells. **f** Percentages of F4/80^+^, PD-L1^+^ and PD-L2^+^ macrophages in colon tissue. **g** Percentages of F4/80^+^ PD-L2^+^ and MR^+^ macrophages. **h** Production of IL-10 in colon tissue under different conditions, as determined by ELISA. **i** Production of IFN-γ in colon tissue under different conditions, as determined by ELISA (n = 5). Each experiment was repeated three times. The data are presented as the means ± SDs of each group (n = 5). *p ≤ 0.05, **p ≤ 0.01, ***p ≤ 0.001, ****p ≤ 0.0001. *ns* not significant. A statistical test was performed via one-way ANOVA, followed by Tukey’s test

As previously demonstrated, glycoproteins are crucial components of TcES that help ameliorate colitis. We aimed to investigate whether M2 macrophage polarization is one of the mechanisms through which these glycoproteins influence colitis development. To evaluate the role of TcES glycans in M2 macrophage generation, we conducted flow cytometry to detect F4/80, PD-L2, PD-L1, and MR in the peritoneal cavity and lamina propria of colitis mice treated with iTcES, TcES depleted of carbohydrates, and TcES depleted of proteins. DSS treatment did not significantly alter the frequency of F4/80^+^PDL2^+^PDL1^+^ cells (Fig. 9a); however, iTcES treatment resulted in an increased percentage of F4/80^+^PDL2^+^PDL1^+^ cells in the peritoneal cavity. In contrast, the depletion of glycans and proteins in TcES reduced M2 macrophage polarization (Fig. 9b). In addition to increasing the frequency of PD-L2- and PD-L1-positive macrophages, iTcES also induced the overexpression of these ligands on the cell membrane, as measured by the mean fluorescence intensity (MFI) (Fig. 9c). Conversely, the absence of carbohydrates and proteins in TcES decreased the PD-L2 MFI in colitis mice (Fig. 9c). However, the PD-L1 MFI remained unaffected by carbohydrate depletion but was reduced by protein depletion in TcES. A similar trend was observed in the colon, where the frequency of PD-L2- and PD-L1-positive cells increased with iTcES treatment but decreased with TcES treatments lacking glycans or proteins (Fig. 9d–f). In addition, the frequency of MR-positive macrophages increased with iTcES and decreased with protein- and carbohydrate-depleted TcES treatment (Fig. 9e, g). Furthermore, the expression of IL-10 in colon tissue strongly depended on glycans and proteins within iTcES (Fig. 9h), reflecting the observed reduction in IFN-γ production (Fig. 9i).

## Discussion

There is evidence that helminths and their secreted products may have therapeutic potential in treating inflammatory diseases such as UC (Fawzy et al. 2024; Alghanmi et al. 2024; Hou et al. 2022; Rawat et al. 2024; Shi et al. 2022). Previous findings indicate that *T. crassiceps* infection induces M2 macrophage polarization and, upon transfer to mice, modulates colitis pathogenesis (Ledesma-Soto et al. 2015). Our results indicate that iTcES confers protection against colitis-associated damage by reducing the clinical manifestations of the disease and ameliorating intestinal mucosal injury through distinct mechanisms. These include increasing goblet cell numbers without concomitant fibrosis, recruiting M2 macrophages, enhancing adherens junction integrity, reducing inflammatory responses, and decreasing oxidative stress. Unlike the corticosteroid dexamethasone, a commonly used treatment for colitis (Acevedo et al. 2024; Liu et al. 2022; Wang et al. 2017), iTcES effectively controls colonic inflammation without apparent hepatic damage, suggesting a potentially improved safety profile. However, other physiologic parameters, such as creatinine and urea levels, may be lacking, limiting our ability to confirm these findings.

Helminth infections are known to increase the number of host goblet cells as a mechanism for expulsion from the intestine (Su et al. 2018). We leveraged this phenomenon here by utilizing iTcES to enhance mucin-producing goblet cells, thereby protecting epithelial cells from colitis-induced disruption. Given that inflammation and epithelial barrier disruption are hallmarks of colitis (Bandyopadhyay et al. 2021; Mansouri et al. 2024; Meng et al. 2024), we investigated the potential of iTcES to modify the colonic proinflammatory microenvironment as an alternative therapeutic approach for treating colitis. iTcES treatment resulted in a significant reduction in TNF-α, IL-1β, IL-23, IL-17F, and IL-33 levels; all of these cytokines are known to exacerbate colitis by promoting intestinal epithelial cell death and disrupting the epithelial barrier (Bandyopadhyay et al. 2021; Dunleavy et al. 2023; Neurath 2024). Considering that anti-TNF-α immunotherapy, while employed in IBD (Melsheimer et al. 2019), is not universally effective and that newer therapies targeting IL-23R or IL-17, which play a detrimental role in colitis (Tang et al. 2018), are costly (Ananthakrishnan et al. 2024; Berger et al. 2024; Mansouri et al. 2024), iTcES represents a promising biotherapeutic alternative. The observed reduction in IL-17 levels following iTcES treatment is consistent with studies demonstrating the detrimental role of IL-17 in colitis (Tang et al. 2018). iTcES also reduced the production of IL-33, a cytokine that is overexpressed in colitis (Palmieri et al. 2021; Pastorelli et al. 2010; Qiu et al. 2020; Schumacher et al. 2024), suggesting reduced damage to colonic tissue. Concurrently, iTcES increased the levels of cytokines such as IL-22, IL-31, and IL-4, which have a protective effect on the colon. IL-22 protects mice from IBD by promoting epithelial cell proliferation, restoring goblet cells, and restoring the expression of tight junction proteins, such as Claudin-1, Zo-1, and E-cadherin (He et al. 2022; Sugimoto et al. 2008). Thus, iTcES-induced IL-22 production coincides with the observed increase in goblet cells and E-cadherin expression. Another cytokine augmented by iTcES treatment is IL-31, which stimulates the proliferation of hematopoietic progenitors in the bone marrow and spleen (Broxmeyer et al. 2007); however, it remains unknown whether iTcES-induced IL-31 may favor hematopoietic progenitors that contribute to colonic epithelial cell replenishment in colitis.

iTcES increased serum MCP-1, possibly driving M2 macrophage recruitment, which is consistent with the known role of MCP-1 in monocyte recruitment to sites of injury and inflammation (Deshmane et al. 2009; Yang et al. 2023). IL-4 modulation by iTcES in colitis and the recently described benefits of IL-4-stimulated (M2) macrophage transfer in colitis and colitis-associated colon cancer (CAC) highlight the therapeutic potential of M2 macrophages (Callejas et al. 2021). iTcES treatment elevated IL-4 production, favoring an M2 macrophage profile in the peritoneum and colon, thereby establishing an anti-inflammatory microenvironment that modulates colitis. This result aligns with that of *Trichinella spiralis* excretory/secretory products, which drive M2 macrophage polarization and attenuate DSS-induced colitis (Wang et al. 2020). iTcES also reduced the recruitment of inflammatory monocytes (CD11b^+^Ly6C^hi^Ly6G^-^) and neutrophils (CD11b^+^Ly6C^-^Ly6G^+^) to the lamina propria, which aligns with their unfavorable roles in colitis and CAC (Cao et al. 2022; Deshmane et al. 2009; Meng et al. 2024; Shin et al. 2023). These findings suggest that iTcES may attenuate colonic inflammation by preventing the influx of neutrophils and inflammatory monocytes.

Neutrophils represent a significant source of reactive oxygen and nitrogen species (ROS, RNS), contributing to inflammation as well as DNA and protein damage (Awasthi and Sarode 2024; Bui et al. 2021; Butin-Israeli et al. 2019; Zeng et al. 2019). Evidence indicates that UC patients exhibit damage to extraintestinal organs, such as the liver (Shen et al. 2021). Therefore, we evaluated ROS production in the colon and liver and detected elevated ROS levels in both organs of DSS-induced colitis mice, as previously reported (Guo et al. 2022; Hwang et al. 2020). Interestingly, iTcES administration inhibited the excess production of ROS in DSS-induced colitis model mice, suggesting that iTcES may have antioxidant properties. ROS react with nitric oxide, which leads to RNS formation, leading to tyrosine nitration, which modifies protein structure and impairs function; 3-nitrotyrosine (3-NT) is a biomarker of oxidative damage in proteins (Bandookwala et al. 2020; Calderón-Torres et al. 2019). Here, we demonstrated that iTcES treatment reduces 3-NT levels in the colon, indicating decreased protein damage. Moreover, exacerbated inflammation induced by DSS promoted the apoptosis of colonic epithelial cells, an effect mitigated by iTcES treatment. Thus, iTcES has anti-inflammatory, antioxidant, and antiapoptotic effects, protecting the liver and colon during acute colitis.

iTcES treatment downregulated nuclear factor-kappa B (NF-κB) and p38 activation in the colon. Considering that these pathways regulate the transcription of inflammatory cytokines involved in colitis (Wei et al. 2021; Xue et al. 2023) and represent therapeutic targets (Hajji et al. 2023; Liu et al. 2023; Ten Hove et al. 2002; Wei et al. 2021; Xue et al. 2023), these findings further support the therapeutic potential of iTcES.

Glycans are important biomolecules that mediate diverse functions and are recognized in helminth-derived products because they play crucial roles in host‒parasite interactions and immune modulation (Dissanayake et al. 2004; Meyer et al. 2007; Van Liempt et al. 2007). Previous studies indicating the potential of carbohydrates to ameliorate DSS-induced colitis (He et al. 2019; Zhang et al. 2022) prompted our investigation into the specific contribution of glycans to the anticolitic activity of TcES. Our findings demonstrate that d-glucose, d-mannose, d-galactose, N-acetylglucosamine, and N-acetylgalactosamine, which are glycans within iTcES, are essential for reversing colitis symptoms, as glycan depletion abrogates the therapeutic efficacy of iTcES, rendering it unable to mitigate damage to colon tissue architecture, including crypt and goblet cell deterioration. Furthermore, iTcES glycans protected the epithelial barrier from DSS-induced injury by increasing E-cadherin and β-catenin expression and decreasing the nuclear translocation of active β-catenin (nonphosphorylated β-catenin) in mice with colitis, thereby enhancing epithelial barrier integrity. In addition, glycans in iTcES are necessary for M2 macrophage polarization.

## Conclusion

In summary, while helminth-derived products can reduce colitis damage, most studies have focused on the role of helminth-derived proteins in modulating inflammation or recruiting different cell populations to the colon. Comparatively, few studies in this field have explored other bioactive components, such as fatty acid derivatives (Wangchuk et al. 2019) and glycans. Therefore, our results provide new insights beyond the ability of iTcES to modulate inflammatory responses, reduce oxidative stress, and mitigate protein damage. Here, we demonstrate that the intact TcES glycan structure is essential for its anticolitic effectiveness and that iTcES promotes the formation of adherens junctions by increasing E-cadherin and β-catenin expression in the colon, both of which are crucial for maintaining epithelial barrier function and intestinal epithelial homeostasis (Lialios and Alimperti 2025). These findings indicate that iTcES exerts a glycan-dependent protective effect in the early stages of DSS-induced colitis.

## Supplementary Information

Below is the link to the electronic supplementary material.

Supplementary file1 Dose‒response test of TcES in a DSS-induced colitis model. To determine the optimal dose of TcES for the colitis model, groups of BALB/c mice with colitis were treated with different concentrations of TcES: (4% DSS + 200 μg of TcES), (4% DSS + 100 μg of TcES), and (4% DSS + 50 μg of TcES). a Body weight loss and DAI (n = 4–6) of the different concentrations of TcES. b Macroscopic appearance of colons and colon length in the dose‒response test of TcES (n = 4–6) (PPTX 13241 KB)

Supplementary file2 Detection of carbohydrates in TcES. The infrared spectra (1450--650 cm-1) of various carbohydrates were compared with the infrared spectra of intact TcES, TcES without carbohydrates (TcES wo/c), and TcES without proteins (TcES wo/p). The red line in all the spectra corresponds to the standard carbohydrate indicated in each graph. a Fingerprint of D-glucose at 20 mg/ml compared with the black line of intact TcES and the green and orange lines of TcES wo/c (two batches). b D-mannose 20 mg/ml fingerprint; the black line corresponds to intact TcES, the green line corresponds to TcES wo/p, and the orange line corresponds to TcES wo/c. c D-galactose fingerprint at 20 mg/ml; the black line illustrates intact TcES, and the green and orange lines denote TcES wo/c (two batches). d Fingerprint of D-fructose at 20 mg/ml; the black line represents intact TcES, while the green and orange lines represent TcES wo/c. e Fingerprint of D-glucose at 500 mg/ml; the black line indicates TcES wo/p. f Fingerprint of D-mannose at 500 mg/ml; the black line indicates TcES wo/p. g Fingerprint of D-galactose at 200 mg/ml; the black line represents TcES wo/p. h Fingerprint of D-fructose at 500 mg/ml; the black line represents TcES wo/p. i Carbohydrate concentrations in different lots of TcES: galactose (24.33 μg/ml), glucose (11.49 μg/ml), and mannose (11.12 μg/ml), according to the respective spectra. The data are presented as the means ± SDs for each group (n = 4 batches) (PPTX 158 KB)

Supplementary file3 In vivo positive effects of TcES treatment on DSS-induced experimental colitis. TcES alleviated the progression of colitis by reducing the proinflammatory environment while promoting an anti-inflammatory response that protects the epithelial barrier by increasing the number of goblet cells and increasing the expression of E-cadherin and β-catenin. In addition, TcES decreased neutrophil infiltration, which may downregulate ROS and 3-NT levels, preventing DNA and protein damage in the colon and liver. Furthermore, TcES promoted an anti-inflammatory microenvironment and promoted M2 macrophage polarization. These effects are lost when the glycan component in TcES is altered (PPTX 368 KB)

## Data Availability

All the data presented here will be made available upon request.
