# Supplementary material for: Intact glycoconjugates from Taenia crassiceps excreted/secreted products ameliorate chemically induced colitis by modulating inflammation and strengthening adherens junctions
Source: Inflammopharmacology. 2025 Jun 27;33(8):4725–47. doi: 10.1007/s10787-025-01821-y (PMC12397184; doi:10.1007/s10787-025-01821-y)
Supplement: Supplementary file 1 — Supplementary file1 Dose‒response test of TcES in a DSS-induced colitis model. To determine the optimal dose of TcES for the colitis model, groups of BALB/c mice with colitis were treated with different concentrations of TcES: (4% DSS + 200 μg of TcES), (4% DSS + 100 μg of TcES), and (4% DSS + 50 μg of TcES). a Body weight loss and DAI (n = 4–6) of the different concentrations of TcES. b Macroscopic appearance of colons and colon length in the dose‒response test of TcES (n = 4–6) (PPTX 13241 KB) [file 10787_2025_1821_MOESM1_ESM.pptx]

## Slide 1
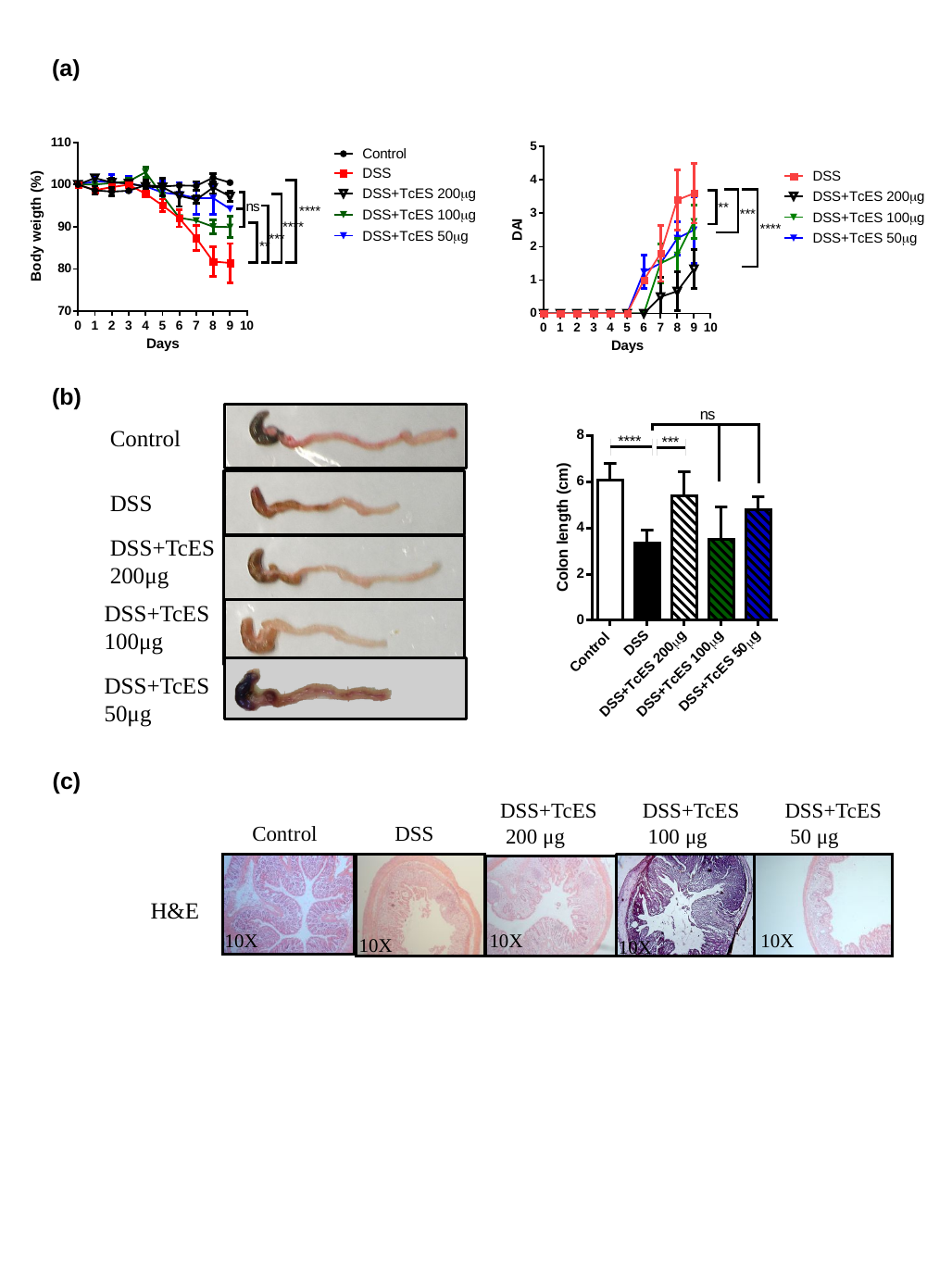

(a)
(b)
Control
DSS
DSS+TcES 200μg
DSS+TcES 100μg
DSS+TcES 50μg
(c)
DSS+TcES
 200 μg
DSS+TcES
 100 μg
DSS+TcES
 50 μg
DSS
Control
H&E
10X
10X
10X
10X
10X
