# Supplementary material for: Intact glycoconjugates from Taenia crassiceps excreted/secreted products ameliorate chemically induced colitis by modulating inflammation and strengthening adherens junctions
Source: Inflammopharmacology. 2025 Jun 27;33(8):4725–47. doi: 10.1007/s10787-025-01821-y (PMC12397184; doi:10.1007/s10787-025-01821-y)
Supplement: Supplementary file 2 — Supplementary file2 Detection of carbohydrates in TcES. The infrared spectra (1450--650 cm-1) of various carbohydrates were compared with the infrared spectra of intact TcES, TcES without carbohydrates (TcES wo/c), and TcES without proteins (TcES wo/p). The red line in all the spectra corresponds to the standard carbohydrate indicated in each graph. a Fingerprint of D-glucose at 20 mg/ml compared with the black line of intact TcES and the green and orange lines of TcES wo/c (two batches). b D-mannose 20 mg/ml fingerprint; the black line corresponds to intact TcES, the green line corresponds to TcES wo/p, and the orange line corresponds to TcES wo/c. c D-galactose fingerprint at 20 mg/ml; the black line illustrates intact TcES, and the green and orange lines denote TcES wo/c (two batches). d Fingerprint of D-fructose at 20 mg/ml; the black line represents intact TcES, while the green and orange lines represent TcES wo/c. e Fingerprint of D-glucose at 500 mg/ml; the black line indicates TcES wo/p. f Fingerprint of D-mannose at 500 mg/ml; the black line indicates TcES wo/p. g Fingerprint of D-galactose at 200 mg/ml; the black line represents TcES wo/p. h Fingerprint of D-fructose at 500 mg/ml; the black line represents TcES wo/p. i Carbohydrate concentrations in different lots of TcES: galactose (24.33 μg/ml), glucose (11.49 μg/ml), and mannose (11.12 μg/ml), according to the respective spectra. The data are presented as the means ± SDs for each group (n = 4 batches) (PPTX 158 KB) [file 10787_2025_1821_MOESM2_ESM.pptx]

## Slide 1
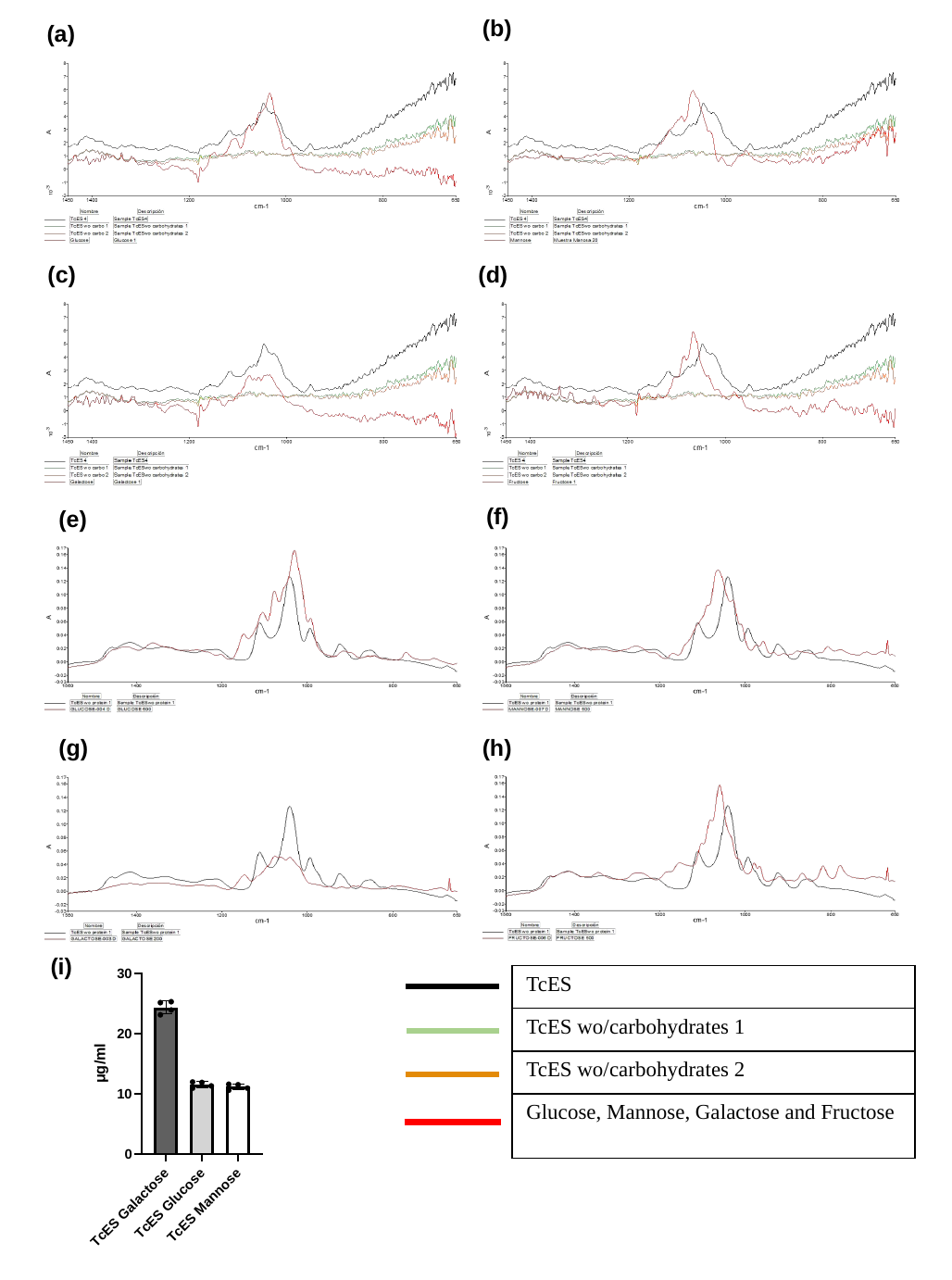

(b)
(a)
(d)
(c)
(f)
(e)
(g)
(h)
(i)
| TcES |
| --- |
| TcES wo/carbohydrates 1 |
| TcES wo/carbohydrates 2 |
| Glucose, Mannose, Galactose and Fructose |
