# Supplementary figures and images for: Intact glycoconjugates from Taenia crassiceps excreted/secreted products ameliorate chemically induced colitis by modulating inflammation and strengthening adherens junctions
Source: Inflammopharmacology. 2025 Jun 27;33(8):4725–47. doi: 10.1007/s10787-025-01821-y (PMC12397184; doi:10.1007/s10787-025-01821-y)

## Slide 1
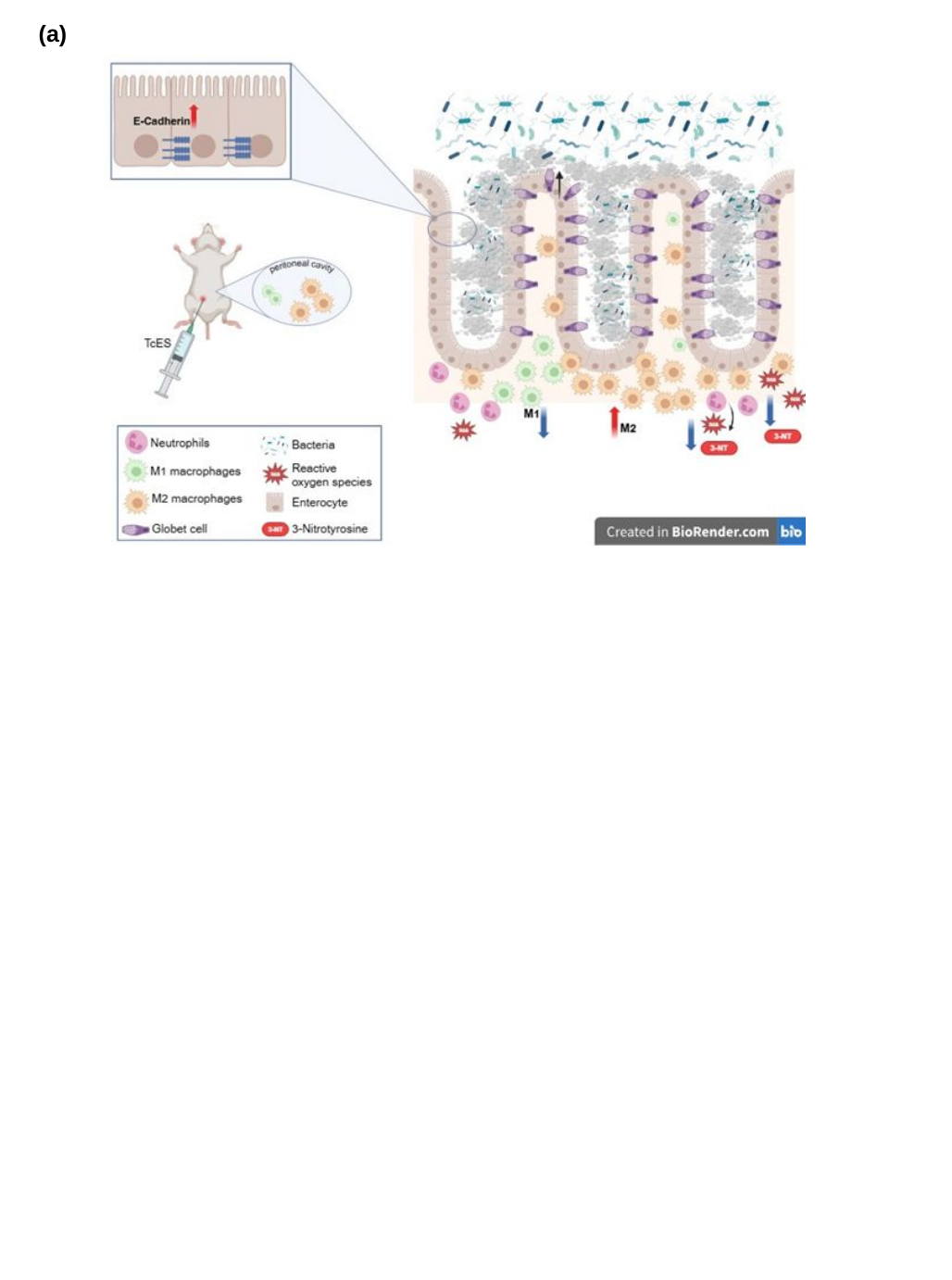

(a)

Supplement: Supplementary file 3 — Supplementary file3 In vivo positive effects of TcES treatment on DSS-induced experimental colitis. TcES alleviated the progression of colitis by reducing the proinflammatory environment while promoting an anti-inflammatory response that protects the epithelial barrier by increasing the number of goblet cells and increasing the expression of E-cadherin and β-catenin. In addition, TcES decreased neutrophil infiltration, which may downregulate ROS and 3-NT levels, preventing DNA and protein damage in the colon and liver. Furthermore, TcES promoted an anti-inflammatory microenvironment and promoted M2 macrophage polarization. These effects are lost when the glycan component in TcES is altered (PPTX 368 KB) [file 10787_2025_1821_MOESM3_ESM.pptx]
